# Implementation of global antimicrobial resistance surveillance system (GLASS) in patients with bacteremia

**DOI:** 10.1371/journal.pone.0190132

**Published:** 2018-01-03

**Authors:** Rujipas Sirijatuphat, Kantarida Sripanidkulchai, Adhiratha Boonyasiri, Pinyo Rattanaumpawan, Orawan Supapueng, Pattarachai Kiratisin, Visanu Thamlikitkul

**Affiliations:** 1 Department of Medicine, Faculty of Medicine Siriraj Hospital, Mahidol University, Bangkok, Thailand; 2 Department of Research and Development, Faculty of Medicine Siriraj Hospital, Mahidol University, Bangkok, Thailand; 3 Department of Microbiology, Faculty of Medicine Siriraj Hospital, Mahidol University, Bangkok, Thailand; Azienda Ospedaliera Universitaria di Perugia, ITALY

## Abstract

The global antimicrobial resistance surveillance system (GLASS) was launched by the World Health Organization (WHO) in 2015. GLASS is a surveillance system for clinical specimens that are sent to microbiology laboratory for clinical purposes. The unique feature of GLASS is that clinical data is combined with microbiological data, and deduplication of the microbiological results is performed. The objective of the study was to determine feasibility and benefit of GLASS for surveillance of blood culture specimens. GLASS was implemented at Siriraj Hospital in Bangkok, Thailand using a locally developed web application program (app) to transfer blood culture specimen data, and to enter clinical data of patients with positive blood culture by infection control nurses and physicians via the app installed in their smart phones. The rate of positive blood culture specimens with true infection was 15.2%. *Escherichia coli* was the most common cause of bacteremia. Secondary bacteremia, primary bacteremia, and central line-associated blood stream infection was observed in 61.8%, 30.6%, and 12.6% of cases, respectively. Sepsis was observed in 56.9% of patients. *E*.*coli* was significantly more common in community-acquired bacteremia, whereas *Klebsiella pneumoniae*, *Pseudomonas aeruginosa*, methicillin-resistant *Staphylococcus aureus*, and *Acinetobacter baumannii* were significantly more common in hospital-acquired bacteremia. Hospital-acquired isolates of *E*.*coli*, *K*.*pneumoniae*, *A*.*baumannii*, *P*.*aeruginosa*, *S*.*aureus* and *Enterococcus faecium* were more resistant to antibiotics than community-acquired isolates. In-hospital mortality was significantly higher in patients with antibiotic-resistant bacteremia than in patients with antibiotic non-resistant bacteremia (40.5% *vs*. 28.5%, *p*<0.001). The patients with antibiotic-resistant bacteremia consumed more resources than those with antibiotic non-resistant bacteremia. Blood culture results combined with patient clinical data were shown to have more benefit for surveillance of antimicrobial resistance, and to be more applicable for developing local antibiotic treatment guidelines for patients suspected of having bacteremia. However, GLASS consumed more time and more resources than the conventional laboratory-based surveillance system.

## Introduction

Antimicrobial resistance (AMR) is a continually evolving public health crisis all over the world. The annual AMR burden in Thailand is estimated to be 100,000 new AMR infections, additional 3 million days of hospital stay, and 30,000 deaths [1]. The annual cost of AMR infections in Thailand is estimated to be US$ 200 million for antibiotics and US$ 13,000 million or 0.6% of GDP for total economic loss [1]. Reported estimated AMR burden figures include hospital-acquired AMR infections, but they do not include community-acquired AMR infections [2–4]. It is projected that there will be 10 million AMR-related deaths each year and a 3% annual reduction in world GDP by 2050 if effective containment of AMR at a global level is not effectively implemented [5].

AMR surveillance is one of the pillars of the World Health Organization’s (WHO) 2015 global action plan on AMR [6]. Similar to the system at our center, a laboratory-based AMR surveillance system is used at most healthcare facilities. Limitations of conventional AMR surveillance include the following: 1) the nature of the grown organism (causative agent, colonizer, or contaminant) is usually undetermined; 2) the source of infection is usually absent; 3) the type of infection (community-acquired or hospital-acquired infection) is usually unavailable; and, 4) data from the same patient who has the same isolated organisms with same antibiotic susceptibility profiles are often duplicated in the annual report of isolated organisms and their antibiotic susceptibility. As such, the laboratory-based AMR surveillance system model does not provide information regarding the extent of AMR in a given population, and the data produced by this system has limited value for developing antibiotic guidelines for patients with a specific type of infection.

Global antimicrobial resistance surveillance system (GLASS) was launched by WHO in 2015, and the manual for early implementation of GLASS in human infections is available [7]. GLASS is a case finding strategy that evaluates priority specimens that are routinely sent to laboratory for clinical purposes. Priority specimen types include blood, urine, feces, and urethral and cervical swabs. The benefit of GLASS is that clinical data is combined with microbiological data, and deduplication of the microbiological results is performed in order to eliminate duplicate copies of repeating data. Another potential benefit is that supplementary clinical information (e.g., morbidity, mortality, and cost), intervention outcomes, and potential drivers of AMR can be collected upon availability of resources. GLASS facilitates the monitoring AMR trends and the development of antibiotic guidelines for specific types of infection may be superior to laboratory-based AMR surveillance. However, GLASS may require more time and resources to collect clinical data, and to combine the clinical data with microbiological data.

Stepwise implementation of GLASS began at Siriraj Hospital in June 2016. Surveillance of blood culture specimens was implemented first, followed by feces, sputum, and urine specimens. The aim of GLASS implementation was to determine the feasibility and benefit of GLASS at a 2,300-bed tertiary care university hospital in Thailand. We reported herein the results of implementing GLASS for surveillance of blood culture specimens in patients who had their blood culture samples collected for clinical purposes.

## Patients and methods

This study protocol was approved by the Siriraj Institutional Review Board (SiIRB), Mahidol University, Thailand. The consent from the study patient was waived by the SiIRB. All blood culture specimens collected from the patients for clinical purposes during July 2016 and February 2017 were included. Blood culture specimens were sent to Department of Microbiology and they were processed according the laboratory standard operating procedures using the bioMérieux BacT/ALERT 3D Microbial Identification System or the BD BACTEC FX blood culture system. The period of incubation of the blood sample was 5 days before it was discarded as negative culture. GLASS was implemented by using a locally developed web application program (app) to transfer blood culture specimen data, and to enter clinical data of patients with positive blood culture by infection control nurses and physicians at the hospital wards via the app installed in their smart phones. The web application program has 4 parts. Part I contains microbiology and demographic data of all patients who had blood specimens collected for culture. The information on all blood culture specimens are transferred from the laboratory every day, and they are managed by trained back office personnel before the results of positive blood culture specimens are sent to the designated infection control nurses and physicians. Part II contains clinical data of patients with positive blood cultures, including the nature of the isolated organism (infection or contaminant), source or site of infection (primary bacteremia, secondary bacteremia, central line-associated blood stream infection or CLABSI), type of infection (community-acquired or hospital-acquired infection), severity of infection (sepsis or non-sepsis), empirical antibiotics being given on the date of blood specimen collection, specific antibiotics given after the culture results were available, and clinical outcomes at the end of antibiotic treatment. These data were collected from patient medical records or from responsible healthcare personnel, and they were entered into the program by infection control nurses and/or physicians via their smart phones. The average time for entering part II information was 10 minutes. Part III contains antibiotic susceptibility results of the isolated bacteria reported by the laboratory that were managed by trained back office personnel. Part IV contains patient outcome data at hospital discharge and hospitalization costs that were transferred from the hospital database.

### Definitions

The isolated bacteria is considered the cause of infection or true bacteremia if the patient has clinical features of infection compatible with the isolated bacteria, has no other causes of those clinical features, and the responsible physician treats such recovered bacteria with antibiotic (s). The isolated bacteria is considered contaminant if the patient has no clinical features of infection or the isolated bacteria usually resides on the skin or environment (e.g., *Bacillus* spp., *Corynebacterium* spp., *Propionibacterium* spp.), and it is not compatible with causing the infection which the patient may have, or the responsible physician does not treat such recovered bacteria with antibiotic which contains activity against such bacteria, or the infection resolves without receiving antibiotic against such isolated bacteria.

Bacteremia in a patient who is hospitalized more than 2 days at Siriraj Hospital or other hospitals prior to admission to Siriraj Hospital or has healthcare-associated conditions (e.g. prior hospitalization within 3 months, prior use of antibiotic within 90 days, resident of long term care facility, chronic hemodialysis) is defined as hospital-acquired infection (HAI), whereas bacteremia in a patient who is hospitalized at Siriraj Hospital within 2 days and has no healthcare-associated conditions (e.g. prior hospitalization within 3 months, prior use of antibiotic within 90 days, resident of long term care facility, chronic hemodialysis) is defined as community-acquired infection (CAI).

Primary bacteremia is defined as bacteremia with unknown source of the bacteria isolated from blood specimen. Secondary bacteremia is defined as bacteremia in a patient with prior localized infection caused by the same bacteria isolated from blood specimen. Central line-associated blood stream infection (CLABSI) is defined as bacteremia in a patient with indwelling central intravascular catheter without other sources of the isolated bacteria from blood specimen.

Sepsis is defined as life-threatening organ dysfunction due to bacteremia with clinical features of organ dysfunction, such as respiration rate more than 22 per minute, alteration of consciousness, systolic blood pressure less than 100 mm Hg.

Concordant empirical antibiotic therapy refers to at least one of the given antibiotics has in vitro activity against the isolated bacteria whereas non-concordant empirical antibiotic therapy refers to none of the given antibiotics has in vitro activity against the isolated bacteria.

The clinical outcomes at the end of antibiotic treatment are classified as cure, superinfection or death. The patient with true bacteremia who received antibiotic (s) is considered cure if all clinical features of infections disappear before or at the end of antibiotic therapy. Superinfection is the infection due to other bacteria from any sites in addition to the bacteria isolated from blood specimen in the patient with true bacteremia and receives antibiotic (s) against the bacteria isolated from blood specimen.

Antibiotic-resistant bacteremia is defined as bacteremia caused by carbapenem-resistant or third-generation cephalosporin-resistant *E*.*coli*, *K*.*pneumoniae*, and other Enterobacteriaceae; carbapenem-resistant *P*.*aeruginosa*; carbapenem-resistant *A*.*baumannii*; fluoroquinolone-resistant *Salmonella* spp.; methicillin-resistant *S*.*aureus* (MRSA); methicillin-resistant coagulase-negative staphylococci (MRCNS); vancomycin-resistant *E*.*faecium*; and, penicillin-non-susceptible *S*.*pneumoniae*.

Deduplication of the same bacterial isolates is performed for all episodes of bacteremia with recovery of two or more isolates of the same bacteria. Only one isolate of the same bacteria with the identical antibiotic susceptibility profile is included in the analysis of the rate of antibiotic resistance to each antibiotic.

### Statistical analysis

The minimum sample size of 865 patients calculated for this study was based on an estimated prevalence of bacteremia of 10%±2% in patients who had blood cultures collected, with a type I error of 5%. Data are presented as number and percentage, mean ± standard deviation, or median. Fisher’s exact test or *χ*2 test was used to compare categorical variables and *t* test to compare quantitative variables. All statistical analyses were performed using either SPSS Statistics or Microsoft Excel. A *p*-value of ≤0.05 was considered statistically significant.

## Results

There were 8,196 blood culture specimens from 2,825 admissions of 2,393 patients, and 1,611 isolates of organisms recovered from all blood culture specimens during July 2016 and February 2017.

The percentage of blood cultures positive for any organisms was 40.2% among 2,393 patients, 39.3% among 2,825 admissions, and 18.7% among 8,196 blood specimens. Organisms isolated from all blood specimens from all admissions of all included patients are shown in Table 1. The most common isolated organism was coagulase-negative *Staphylococcus* spp. (CNS), followed by *E*.*coli*, *K*.*pneumoniae*, *S*.*aureus*, *A*.*baumannii*, and *P*.*aeruginosa*.

**Table 1:** Organisms isolated from all blood specimens from all admissions of all included patients.

Type of organism	Number of specimens with positive culture (n = 1,530)	Number of admissions with positive culture (n = 1,109)^a^	Number of patients with positive culture (n = 963)^a^
Coagulase-negative *Staphylococcus* spp.	291 (19.0%)	241 (21.7%)	218 (22.6%)
*Escherichia coli*	276 (18.0%)	226 (20.4%)	193 (20.0%)
*Klebsiella pneumoniae*	166 (10.8%)	131 (11.8%)	117 (12.1%)
*Staphylococcus aureus*	149 (9.7%)	95 (8.6%)	89 (9.2%)
*Acinetobacter baumannii*	111 (7.3%)	72 (6.5%)	70 (7.3%)
*Pseudomonas aeruginosa*	105 (6.9%)	78 (7.0%)	75 (7.8%)
Yeasts^b^	51 (3.3%)	37 (3.3%)	34 (3.5%)
*Proteus mirabilis*	39 (2.5%)	33 (3.0%)	30 (3.1%)
*Enterobacter* spp.	38 (2.5%)	21 (1.9%)	21 (2.2%)
*Enterococcus faecium*	38 (2.5%)	31 (2.8%)	31 (3.2%)
*Enterococcus faecalis*	35 (2.3%)	30 (2.7%)	28 (2.9%)
*Salmonella* spp.	28 (1.8%)	21 (1.9%)	20 (2.1%)
*Stenotrophomonas maltophilia*	27 (1.8%)	19 (1.7%)	19 (2.0%)
*Aeromonas* spp.	26 (1.7%)	20 (1.8%)	18 (1.9%)
Gram-negative rods, NF	21 (1.4%)	20 (1.8%)	18 (1.9%)
Streptococci, group D	21 (1.4%)	20 (1.8%)	14 (1.5%)
Coryneform bacteria	19 (1.2%)	17 (1.5%)	17 (1.8%)
Streptococci, beta-hemolytic	18 (1.2%)	17 (1.5%)	14 (1.5%)
Streptococci, alpha-hemolytic	15 (1.0%)	12 (1.1%)	12 (1.2%)
*Bacillus* spp.	14 (0.9%)	13 (1.2%)	13 (1.3%)
*Micrococcus* spp.	14 (0.9%)	14 (1.3%)	14 (1.5%)
Other Gram-negative bacteria^c^	72 (4.7%)	49 (4.4%)	46 (4.8%)
Other Gram-positive bacteria^d^	24 (1.6%)	22 (2.0%)	21 (2.2%)

^a^ may have had more than one positive culture specimen

^b^ *C*.*tropicalis* (20); *C*.*albicans* (18); *C*.*parapsilosis* complex (4); *Cryptococcus neoformans* (4); *C*.*glabrata* (3); *C*.*guilliermondii* (1); and, *Pseudozyma* spp. (1)

^c^ *Pseudomonas* spp. (11); *Moraxella* spp. (9); *Acinetobacter* spp. (7); *Serratia marcescens* (7); *Achromobacter* spp. (6); *Vibrio* spp. (5); *Burkholderia cepacia* (4); *Haemophilus* spp. (4); *Burkholderia pseudomallei* (3); *Chryseobacterium* spp. (3); *Citrobacter* spp. (3); *Pastuerella* spp. (2); *Capnocytophaga* spp. (1); *Klebsiella oxytoca* (1); *Methylobacterium* spp. (1); *Plesiomonas shigelloides* (1); *Proteus vulgaris* (1); *Providentia rettgeri* (1); and, *Shewanella* spp. (1)

^d^ *Enterococcus* spp. (8); *Streptococcus pneumoniae* (7); *Streptococcus suis* (4); *Aerococcus* spp. (2); *Lactococcus* spp. (1); *Lactobacillus* spp. (1); *Peptostreptococcus* spp. (1)

The percentage of blood cultures positive for bacteria was 38.8% among 2,393 patients, 37.9% among 2,825 admissions, and 18.0% among 8,196 blood specimens. The causative and contaminant bacteria isolated from all blood specimens from all admissions of all included patients are shown in Table 2. The contamination rate of blood cultures was 3.5% among all blood culture specimens and 18.9% among positive blood culture specimens. The most common contaminant was CNS (86.5%). Among all isolates of CNS in blood cultures, 84.9% were contaminants and 15.1% were causative bacteria. CNS isolates that were contaminants tended to be more resistant to antibiotics than CNS causing infections. The percentage of blood cultures with causative agents was 15.2%, with *E*.*coli* being the most common cause of bacteremia, followed by *K*.*pneumoniae*, *S*.*aureus*, *P*.*aeruginosa*, and *A*.*baumannii*.

**Table 2:** Causative and contaminant bacteria isolated from all blood specimens from all admissions of all included patients.

Type of bacteria	Causative bacteria		Contaminant bacteria	
	Number of admissions (n = 775)^a^	Number of patients (n = 728)^a^	Number of admissions (n = 251)^a^	Number of patients (n = 237)^a^
*Escherichia coli*	202 (26.1%)	193 (26.5%)	0 (0%)	0 (0%)
*Klebsiella pneumoniae*	121 (15.6%)	117 (16.1%)	0 (0%)	0 (0%)
*Staphylococcus aureus*	90 (11.6%)	88 (12.1%)	1 (0.4%)	1 (0.4%)
*Pseudomonas aeruginosa*	75 (9.7%)	74 (10.2%)	1 (0.4%)	1 (0.4%)
*Acinetobacter baumannii*	69 (8.9%)	69 (9.5%)	1 (0.4%)	1 (0.4%)
*Enterococcus faecium*	30 (3.9%)	30 (4.1%)	1 (0.4%)	1 (0.4%)
*Proteus mirabilis*	30 (3.9%)	29 (4.0%)	1 (0.4%)	1 (0.4%)
*Enterococcus faecalis*	28 (3.6%)	28 (3.8%)	0 (0%)	0 (0%)
Coagulase-negative *Staphylococcus* spp.	26 (3.4%)	25 (3.4%)	217 (86.5%)	213 (89.9%)
*Enterobacter* spp.	21 (2.7%)	21 (2.9%)	0 (0%)	0 (0%)
Other Gram-negative bacteria	117 (15.1%)	115 (15.8%)	4 (1.6%)	4 (1.7%)
Other Gram-positive bacteria	69 (8.9%)	67 (9.2%)	41 (16.3%)	41 (17.3%)

^a^ may have had more than one type of bacteria

Secondary bacteremia was observed in 61.8% of infection episodes, with primary bacteremia and CLABSI being observed in 30.6% and 12.6% of infection episodes, respectively. Among 479 episodes of secondary bacteremia, the sources of bacteremia were genitourinary tract (37.2%), respiratory tract (24.6%), gastrointestinal tract (23.0%), and musculoskeletal system (10.6%). Among 98 episodes of CLABSI, CNS (21.4%) was the most common bacteria, followed by *S*.*aureus* (20.4%), *A*.*baumannii* (14.3%), *P*.*aeruginosa* (13.3%), *K*.*pneumoniae* (11.2%), *Enterococcus faecium* (9.2%), and *E*.*coli* (6.1%). Sepsis was observed in 56.9% of patients.

Comparisons between bacteremia patients with community-acquired infection (CAI) and bacteremia patients with hospital-acquired infection (HAI) are shown in Table 3. Secondary bacteremia was significantly more prevalent in patients with CAI. Patients with HAI were younger and had more septic episodes. Prevalence of bacteremia due to *K*.*pneumoniae*, *P*.*aeruginosa*, and *A*.*baumannii* was significantly more common in HAI. Overall prevalence of ceftriaxone-resistant *E*.*coli* and *K*.*pneumoniae* was 19.3% in CAI, compared with 69.5% in HAI (*p*<0.001). Methicillin-resistant *S*.*aureus* (MRSA) was significantly more common in hospital-acquired *S*.*aureus* bacteremia, when compared with community-acquired *S*.*aureus* bacteremia (43.2% *vs*. 0%, *p*<0.001). Concordant empirical antibiotic therapy and clinical response of infections at the end of treatment were significantly more favorable in CAI. Length of hospital stay and in-hospital mortality was significantly higher in HAI than in CAI (both *p*<0.001).

**Table 3:** Comparisons between bacteremia patients with community-acquired infection and bacteremia patients with hospital-acquired infection.

Characteristic	Community-acquired infection (CAI)	Hospital-acquired infection (HAI)	*p*-value
Age (years)			
Mean±SD	61.6±21.3	50.5±27.2	<0.001
Median	65	57	
Male gender	168/314 (53.5%)	183/321 (57.0%)	0.37
Organism			
*Escherichia coli*	118/423 (27.9%)	86/373 (23.1%)	0.12
*Klebsiella pneumoniae*	51/423 (12.1%)	72/373 (19.3%)	0.005
*Staphylococcus aureus*	50/423 (11.8%)	39/373 (10.4%)	0.54
*Pseudomonas aeruginosa*	32/423 (7.6%)	44/373 (11.7%)	0.04
*Acinetobacter baumannii*	12/423 (2.8%)	57/373 (15.3%)	<0.001
Clinical features			
Primary bacteremia	121/423 (28.6%)	119/373 (31.9%)	0.31
Secondary bacteremia	298/423 (70.4%)	181/373 (48.5%)	<0.001
Sepsis	216/423 (51.1%)	218/373 (58.4%)	0.04
Empirical antibiotic treatment			
Concordant antibiotic therapy	341/431 (79.1%)	240/382 (62.8%)	<0.001
Non-concordant antibiotic therapy	90/431 (20.9%)	142/382 (37.2%)	<0.001
Length of hospital stay (days)			
Mean±SD	17.9±20.0	43.6±41.0	<0.001
Median	13	29	
Clinical response at the end of treatment			
Response	242/333 (72.7%)	201/349 (57.6%)	<0.001
Superimposed infection	43/333 (12.9%)	67/349 (19.2%)	0.03
Death	48/333 (14.4%)	81/349 (23.2%)	<0.001
In-hospital mortality	79/333 (23.7%)	141/349 (40.4%)	<0.001

Comparisons between patients with primary bacteremia and secondary bacteremia are shown in Table 4. *E*.*coli* and *A*.*baumannii* were significantly more common in secondary bacteremia, whereas *K*.*pneumoniae* was significantly more common in primary bacteremia. Primary bacteremia was significantly more prevalent in HAI. Concordant empirical antibiotic therapy was significantly more common in primary bacteremia. Length of hospital stay, clinical response of infections at the end of treatment, and in-hospital mortality were comparable between secondary bacteremia and primary bacteremia patients. Patients with CLABSI received more frequent non-concordant empirical antibiotic therapy, and they had a significantly longer length of hospital stay.

**Table 4:** Comparisons between patients with primary bacteremia and patients with secondary bacteremia.

Characteristic	Primary bacteremia	Secondary bacteremia	*p*-value
Age (years)			
Mean±SD	51.7±28.8	61.9±19.5	<0.001
Median	57.5	65.0	
Male gender	112/188 (59.6%)	182/333 (54.7%)	0.28
Isolated bacteria			
*Escherichia coli*	48/237 (20.3%)	149/465 (32.0%)	0.001
*Klebsiella pneumoniae*	50/237 (21.1%)	61/465 (13.1%)	0.006
*Staphylococcus aureus*	23/237 (9.7%)	48/465 (10.3%)	0.80
*Pseudomonas aeruginosa*	21/237 (8.9%)	41/465 (8.8%)	0.98
*Acinetobacter baumannii*	11/237 (4.6%)	45/465 (9.7%)	0.02
Clinical features			
Community-acquired infection	128/237 (54.0%)	295/465 (63.4%)	0.02
Hospital-acquired infection	111/237 (46.8%)	166/465 (35.7%)	0.004
Sepsis	142/237 (59.9%)	255/465 (54.8%)	0.20
Empirical antibiotic treatment			
Concordant antibiotic therapy	179/228 (78.5%)	305/444 (68.7%)	0.007
Non-concordant antibiotic therapy	49/228 (21.5%)	139/444 (31.3%)	0.007
Length of hospital stay (days)			
Mean±SD	27.0±34.2	28.6±30.4	0.58
Median	18	19	
Clinical response at the end of treatment			
Response	125/204 (61.3%)	281/444 (63.3%)	0.59
Superimposed infection	32/204 (15.7%)	72/444 (16.2%)	0.86
Death	47/204 (23.0%)	91/444 (20.5%)	0.46
In-hospital mortality	70/204 (34.3%)	148/444 (33.3%)	0.81

Comparisons between bacteremia patients with and without sepsis are shown in Table 5. *K*.*pneumoniae* was more common in bacteremia patients with sepsis. Clinical response of infections at the end of treatment in bacteremia patients with sepsis was less favorable than in bacteremia patients without sepsis. In-hospital mortality in bacteremia patients with sepsis was significantly higher than in bacteremia patients without sepsis.

**Table 5:** Comparisons between bacteremic patients with and without sepsis.

Characteristic	Sepsis	No sepsis	*p*-value
Age (years)			
Mean±SD	56.8±23.1	56.3±25.0	0.80
Median	61	61	
Male gender	171/321 (53.3%)	145/263 (55.1%)	0.68
Organism			
*Escherichia coli*	117/435 (26.9%)	86/361 (23.8%)	0.33
*Klebsiella pneumoniae*	78/435 (17.9%)	46/361 (12.7%)	0.04
*Staphylococcus aureus*	51/435 (11.7%)	40/361 (11.1%)	0.82
*Pseudomonas aeruginosa*	47/435 (10.8%)	29/361 (8.0%)	0.23
*Acinetobacter baumannii*	43/435 (9.9%)	29/361 (8.0%)	0.39
Clinical features			
Community-acquired infection	225/435 (51.7%)	198/361 (54.8%)	0.38
Hospital-acquired infection	220/435 (50.6%)	168/361 (46.5%)	0.27
Primary bacteremia	142/435 (32.6%)	99/361 (27.4%)	0.12
Secondary bacteremia	265/435 (60.9%)	214/361 (59.3%)	0.64
Empirical antibiotic treatment			
Concordant antibiotic therapy	334/463 (72.1%)	255/367 (69.5%)	0.40
Non-concordant antibiotic therapy	129/463 (27.9%)	112/367 (30.5%)	0.40
Length of hospital stay (days)			
Mean±SD	32.5±37.5	29.7±30.0	0.36
Median	22	21	
Clinical response at end of treatment			
Response	215/377 (57.0%)	213/278 (76.6%	<0.001
Superimposed infection	62/377 (16.5%)	44/278 (15.8%)	0.83
Death	100/377 (26.5%)	21/278 (7.6%)	<0.001
In-hospital mortality	154/377 (40.9%)	54/278 (19.4%)	<0.001

Comparisons between patients who received concordant empirical antibiotics and patients who received non-concordant empirical antibiotics are shown in Table 6. Patients with *S*.*aureus* bacteremia, community-acquired infection, and primary bacteremia received concordant empirical antibiotics more often; whereas, patients with *A*.*baumannii* bacteremia, hospital-acquired bacteremia, and secondary bacteremia received non-concordant empirical antibiotics more often. Patients who received non-concordant empirical antibiotics had a longer length of hospital stay and higher in-hospital mortality than patients who received concordant empirical antibiotics.

**Table 6:** Comparisons between patients who received concordant empirical antibiotic therapy and patients who received non-concordant empirical antibiotic therapy.

Characteristic	Concordant empirical therapy*	Non-concordant empirical therapy**	*p*-value
Age (years)			
Mean±SD	55.1±25.5	56.4±26.1	0.57
Median	59	63	
Male gender	209/405 (51.6%)	100/161 (62.1%)	0.02
Organism			
*Escherichia coli*	152/565 (26.9%)	49/223 (22%)	0.17
*Klebsiella pneumoniae*	90/565 (15.9%)	31/223 (13.9%)	0.51
*Staphylococcus aureus*	73/565 (12.9%)	16/223 (7.2%)	0.02
*Pseudomonas aeruginosa*	46/565 (8.1%)	28/223 (12.6%)	0.05
*Acinetobacter baumannii*	25/565 (4.4%)	44/223 (19.7%)	<0.001
Clinical features			
Community-acquired infection	342/565 (60.5%)	94/223 (42.3%)	<0.001
Hospital-acquired infection	224/565 (39.6%)	139/223 (62.3%)	<0.001
Primary bacteremia	186/565 (32.9%)	51/223 (22.9%)	0.006
Secondary bacteremia	323/565 (57.2%)	156/223 (70.0%)	<0.001
Sepsis	324/565 (57.3%)	118/223 (52.9%)	0.27
Length of hospital stay (days)			
Mean±SD	32.9±38.8	41.3±33.5	0.02
Median	20	31	
Clinical response at end of treatment			
Response	294/462 (63.6%)	103/223 (46.2%)	<0.001
Superimposed infection	78/462 (16.9%)	46/223 (20.6%)	0.23
Death	90/462 (19.5%)	74/223 (33.2%)	<0.001
In-hospital mortality	134/462 (29.0%)	110/223 (49.3%)	<0.001

Duplicate bacterial isolates with identical antibiotic susceptibility profiles for each episode of bacteremia were observed in 80 out of 216 isolates of *E*.*coli*, 45 out of 177 isolates of *K*.*pneumoniae*, 25 out of 112 isolates of *A*.*baumannii*, 25 out of 108 isolates of *P*.*aeruginosa*, 5 out of 28 isolates of *Salmonella* spp., 7 out of 36 isolates of *E*.*faecalis*, 6 out of 38 isolates of *E*.*faecium*, 45 out of 311 isolates of CNS, and 49 out of 149 isolates of *S*.*aureus*. Comparisons of antibiotic susceptibility profiles between non-duplicate isolates and duplicate isolates of the aforementioned bacteria revealed no significant differences in antibiotic susceptibility between non-duplicate isolates and duplicate isolates.

Comparisons of antibiotic susceptibility of common or important antibiotic-resistant bacteria between community-acquired and hospital-acquired bacterial isolates are shown in Table 7. Hospital-acquired *E*.*coli* and *K*.*pneumoniae* isolates were more resistant to ceftriaxone than community-acquired isolates. Hospital-acquired *K*.*pneumoniae*, *A*.*baumannii*, *P*.*aeruginosa* isolates were more resistant to meropenem and piperacillin-tazobactam than community-acquired isolates. None of community-acquired *S*.*aureus* isolates were MRSA whereas 43% of hospital-acquired *S*.*aureus* isolates were MRSA. None of community-acquired *E*.*faecuim* isolates were resistant to vancomycin whereas 29% of hospital-acquired *E*.*faecuim* isolates were resistant to vancomycin.

**Table 7:** Percentage of antibiotic susceptibility of common or important community-acquired bacterial isolates (CABI) and hospital-acquired bacterial isolates (HABI).

Bacteria		Number of isolates	Amoxicillin/clavulanate	Piperacillin/tazobactam	Ceftazidime	Ceftriaxone	Cefepime	Meropenem	Amikacin	Ciproflo-xacin	Colistin	Oxacillin	Vancomycin
*Escherichia coli*	CABI	122	85	99	91	73	94	100	100	58			
	HABI	94	63	92	36	18	63	100	98	39			
*Klebsiella pneumoniae*	CABI	52	94	90	92	98	96	96	98	88			
	HABI	80	53	56	17	44	59	73	80	49			
*Acinetobacter baumannii*	CABI	12		64	64	0	55	70	80	55	100		
	HABI	75		24	25	0	25	25	34	25	100		
*Pseudomonas aeruginosa*	CABI	34		91	91		91	85	97	82	100		
	HABI	49		67	67		67	63	73	73	98		
*Staphylococcus aureus*	CABI	56								95		100	100
	HABI	44								57		57	100
*Enterococcus faecium*	CABI	8											100
	HABI	24											71

Many bacteria on the list announced by WHO for the antibiotic-resistant bacteria considered posing the greatest threat to human health [8] were observed in this study. Carbapenem-resistant *E*.*coli* was observed in 1.3% of *E*.*coli* isolates; carbapenem-resistant *K*.*pneumoniae* in 20.0% of *K*.*pneumoniae* isolates; carbapenem-resistant *P*.*aeruginosa* in 27.7% of *P*.*aeruginosa* isolates; and, carbapenem-resistant *A*.*baumannii* in 69.5% of *A*.*baumannii* isolates. Vancomycin-resistant enterococci (VRE) were found in 14.3% of *Enterococcus* spp. isolates. All VRE isolates were *E*.*faecium*. MRSA was isolated from 19.0% of all *S*.*aureus* isolates. MRSA bacteremia was 0% in community-acquired *S*.*aureus* bacteremia, but was 43% in hospital-acquired *S*.*aureus* bacteremia.

Median length of hospital stay in all hospitalized patients with true bacteremia was 17 days. Overall in-hospital mortality of patients with true bacteremia was 33.3%. Mortality was significantly higher in patients with antibiotic-resistant bacteremia than in patients with antibiotic-non-resistant bacteremia (40.5% *vs*. 28.5%, *p*<0.001) as shown in Table 8. The mortality attributable to AMR was 12.0% (95% Confidence Interval 5.7% to 18.1%). Patients with antibiotic-resistant *A*.*baumannii* or *E*.*faecium* bacteremia had the highest mortality (66.7%). Based on these findings from the data collected for eight months, the estimated annual number of deaths was 194 patients with antibiotic-resistant bacteremia, and 171 patients with antibiotic-non-resistant bacteremia.

**Table 8:** In-hospital mortality of patients with priority pathogens as the cause of bacteremia.

Bacteria	Mortality		*p*-value
	Antibiotic-resistant bacteria^a^	Antibiotic-non-resistant bacteria	
*Escherichia coli*	23/106 (21.7%)	18/100 (18.0%)	0.51
*Klebsiella pneumoniae*	23/45 (51.1%)	22/65 (33.8%)	0.07
Other Enterobacteriaceae	3/8 (37.5%)	12/43 (27.9%)	0.58
*Pseudomonas aeruginosa*	10/21 (47.6%)	19/47 (40.4%)	0.58
*Acinetobacter baumannii*	38/57 (66.7%)	3/24 (12.5%)	<0.001
*Salmonella* spp.	0/2 (0%)	1/2 (50.0%)	0.25
*Staphylococcus aureus*	9/16 (56.3%)	13/47 (27.7%)	0.04
Coagulase-negative *Staphylococcus* spp.	1/14 (7.1%)	-	-
*Enterococcus faecium*	6/9 (66.7%)	12/20 (60.0%)	0.73
*Streptococcus pneumoniae*	0/1 (0%)	0/3 (0%)	-
Overall in-hospital mortality	113/279 (40.5%)	100/351 (28.5%)	<0.001

^a^ Carbapenem-resistant or third-generation cephalosporin-resistant *E*.*coli*, *K*.*pneumoniae*, and other Enterobacteriaceae; carbapenem-resistant *P*.*aeruginosa*; carbapenem-resistant *A*.*baumannii*; fluoroquinolone-resistant *Salmonella* spp.; MRSA; MRCNS; vancomycin-resistant *E*.*faecium*; and, penicillin-non-susceptible *S*.*pneumoniae*

The cost of hospitalization for each patient with bacteremia was retrieved from the hospital database of the Computer Unit and the Financial Department of Siriraj Hospital. The annual cost of hospitalizations for patients with bacteremia was estimated from the cost of hospitalizations for patients with bacteremia during the study period for eight months. The estimated total annual cost of hospitalizations for patients with bacteremia was US$ 10,854,132, of which US$ 5,409,816 was spent for patients with antibiotic-non-resistant bacteremia (US$ 8,614/admission), and US$ 5,444,316 was spent for patients with antibiotic-resistant bacteremia (US$ 15,379/admission). The estimated annual cost of hospitalizations for patients with community-acquired bacteremia was US$ 2,088,260 (US$ 4,725/admission) and hospital-acquired bacteremia was US$ 8,765,872 (US$ 16,233/admission).

## Discussion

The manual for early implementation of GLASS in human infections recommends blood, urine, feces, and urethral and cervical swabs as priority specimens; and *E*.*coli*, *K*.*pneumoniae*, *A*.*baumannii*, *S*.*aureus*, *S*.*pneumoniae*, *Salmonella* spp., *Shigella* spp., and *N*.*gonorrhoeae* as priority bacteria. However, GLASS implementation at Siriraj Hospital included collection of sputum because respiratory tract infection is very common infection in hospitalized patients [9, 10]. Furthermore, the interpretation of sputum culture results is challenging, regardless of whether the isolated organism is causative agent, colonizer, or contaminant. We did not include urogenital swabs for gonococcal culture, because these specimens are very uncommon. We also collected *P*.*aeruginosa* because it is one of the most common causative bacteria, especially in HAI [9, 10].

A key feature of GLASS is that patient clinical data and microbiological data are combined. We recognize that many types of relevant patient clinical data are often not included in the information submitted to the laboratory along with the clinical sample. Moreover, new and important patient clinical data will become available after the clinical sample has been sent to the laboratory. As a result, the report of the culture result is usually incomplete and limited in value, because it does not include or take into account these important pieces of missing data. Many clinical data were collected from patients in our study using a locally developed user-friendly web application program that could be installed in a smart phone and conveniently used in patient care areas. Supplementary information that was collected included source of infection, severity of infection, empirical and specific antibiotic therapy, clinical outcomes of infection, patient mortality, and cost of hospitalization. This additional data was important to understand the epidemiology of bacteremia at our center, to enhance our ability to develop more appropriate local antibiotic guidelines, and to estimate health and economic burden of bacteremia caused by AMR bacteria.

The reported blood culture contamination rate of 3.5% was higher than the acceptable target rate of less than 3% [11]. CNS was the most common blood culture contaminant, and it accounted for 19% of positive blood culture specimens, but it was less than 32% of CNS among all isolated bacteria in the 2016 annual report of the National Antimicrobial Resistance Surveillance Centre, Thailand [12]. The rate of blood culture contamination is a recommended indicator of health care quality. We, therefore, intend to implement additional measures to reduce the rate of contaminants in blood cultures. Our study revealed that 15.1% of CNS isolated from blood specimens were causative bacteria based on patient clinical data. Therefore, information relating to clinical features of patients with positive blood culture for CNS was extremely important for determining if CNS was a causative agent that required antibiotic therapy. This observation emphasized the importance and value of collecting clinical data in addition to demographic data recommended in GLASS manual. CNS that caused infection tended to be less resistant to antibiotics than CNS that was contaminant. This observation suggested that bacteria isolated from sputum and urine samples that was colonizer might be more resistant to antibiotics than isolated bacteria that was the cause of infection.

We would have been unable to determine if isolated bacteria was primary bacteremia, secondary bacteremia, or CLABSI unless the clinical features of patients with positive blood cultures were taken into account. Classification of these 3 categories of bacteremia was necessary, because the bacteria that caused different types of bacteremia were different, and each type of bacteremia required a different regimen of empirical antibiotic therapy. If all bacteria that cause all types of bacteremia would have been combined and those data were used for developing empirical antibiotic guidelines for patients with bacteremia, many patients with different types of bacteremia would have received inappropriate antibiotic regimens.

Differentiation between bacteremia in CAI and HAI was necessary since the types of causative bacteria, the antibiotic susceptibility profiles of bacteria, and the clinical outcomes of CAI and HAI were significantly different. Important data including hospitalization at other healthcare facilities >2 days within 90 days, healthcare-associated conditions, and duration of current hospitalization >2 days were needed to determine if bacteremia was HAI. We found that if duration from date of hospitalization to date of blood culture collection of ≤2 days was used to classify bacteremia as CAI, at least 10% of patients with HAI would have been classified as CAI. Misclassification of some bacteremic episodes as CAI instead of HAI resulted in significantly higher prevalence of antibiotic resistance to causative bacteria, such as prevalence of community-acquired MRSA from 0% to 9% and prevalence of ceftriaxone-resistant *E*.*coli* and *K*.*pneumoniae* from 19.3% to 38.5%.

Observations from our study confirmed the results of previous studies that *E*.*coli*, *K*.*pneumoniae*, *S*.*aureus*, *A*.*baumannii*, and *P*.*aeruginosa* were common causative agents of bacteremia, and that the clinical outcomes of bacteremia due to antibiotic-resistant bacteria were unfavorable [1, 9, 10, 13–19]. However, our study results revealed additional important details about bacteremia, such as the sources of secondary bacteremia, sepsis status in bacteremia patients, and antibiotic susceptibility of community-acquired and hospital-acquired bacterial blood isolates–all of which will be useful for managing patients with suspected bacteremia in the future.

We found no significant differences in antibiotic susceptibility between non-duplicate isolates and duplicate isolates among *E*.*coli*, *K*.*pneumoniae*, *Salmonella* spp., *A*.*baumannii*, *P*.*aeruginosa*, *E*.*faecalis*, *E*.*faecium*, *S*.*aureus*, CNS, and *S*.*pneumoniae*. This could be due to the fact that most patients had only one episode of bacteremia, each episode of bacteremia usually had one type of bacteria, and most isolated bacteria were causative agents. It is anticipated that differences in antibiotic susceptibility between non-duplicate and duplicate isolates of bacteria from other clinical specimens commonly colonized with organisms (e.g., sputum and urine) should be observed similar to our finding of more antibiotic resistance in CNS isolates that were contaminants than CNS isolates that were the cause of infection.

Many metrics of AMR surveillance according to GLASS protocol are presented in our results. However, some recommended metrics could not be computed, such as the number of blood cultures per 100,000 inhabitants. This is because many patients who receive medical care from Siriraj Hospital are not residents of Siriraj Hospital catchment areas. Siriraj Hospital is a national tertiary referral hospital and we receive and treat patients that are referred from across Thailand.

Based on our findings, GLASS was superior to the laboratory-based surveillance for blood culture specimens in patients with bacteremia. Although GLASS consumed more time and resources than the laboratory-based surveillance system, the data derived from GLASS was more useful for developing antibiotic guidelines for patients suspected of having bacteremia. Data derived from GLASS are also valuable for estimating and monitoring the antimicrobial consumption and usage, and health and economic burden of AMR. Furthermore, the results from GLASS can be used to estimate and monitor a drug resistance index [20]. Given that the GLASS that we implemented at Siriraj Hospital exceeded the minimum recommended criteria set forth in GLASS manual, it may be difficult to fully and permanently implement this system in the near term on institution-wide basis. Therefore, we may activate GLASS for one 6-month period every other year because the types of causative agents of infections and their antibiotic susceptibility should not have dramatic changes over a short period of time. Another alternative for ideal implementation of GLASS would require responsible personnel who will send the clinical specimen for culture to provide all relevant patient clinical data along with request for culture of clinical specimen.
